# Commentary: Bibliometric and visualized analysis of 2011–2020 publications on physical activity therapy for diabetes

**DOI:** 10.3389/fmed.2024.1525801

**Published:** 2024-12-24

**Authors:** Yutian Chen, Baitian Fu, Jianqiao Fang

**Affiliations:** ^1^Key Laboratory of Acupuncture and Neurology of Zhejiang Province, The Third Clinical Medical College, Zhejiang Chinese Medical University, Hangzhou, China; ^2^The Third Clinical Medical School, Zhejiang Chinese Medicine University, Hangzhou, China

**Keywords:** physical activity, exercise, diabetes, bibliometric analysis, CiteSpace

## Introduction

We read with interest the article by Huang et al. (1), in which the authors aimed to investigate the global emerging trends in physical activity therapy for diabetes based on a bibliometric analysis of the publications. Their results suggest that “*physical activity therapy for diabetes*” has been accepted remarkably over the last 10 years. The keywords of “impaired glucose tolerance,” “*Cardiovascular outcome*,” “*improves glycemic control*,” “*Self-management*,” and exercise type including “*Aerobic exercise, muscle strength*” may be the latest research frontiers.

The bibliometric study aims to systematically evaluate the current state, research focal points, and emerging trends related to one subject in the past (2). By analyzing the literature using bibliometric methods, we can identify significant findings and provide insights for future research directions (3). In bibliometrics, co-occurrence mapping is used to reveal research hotspots, display the structure of a discipline, and show collaborative relationships, thereby helping understand the overall development of the field (4). There are similar bibliometric articles related to diabetes (5) and others unrelated to diabetes (6), each describing co-occurrence maps where the meaning of each solid circle varies. Unfortunately, this article contains some errors in the description of these maps. Specifically, in the co-occurrence maps of institutions, authors, keywords, and references, each solid circle does not always represent a country or region. Rather, the meaning of the circles differs across different types of maps. For example, in the author co-occurrence map, each solid circle represents an author; However Huang et al. incorrectly stated that the solid circles represent countries and regions in their description of the author co-occurrence map.

## Discussion

The Bibliometric and Visualized Analysis of 2011–2020 Publications on Physical Activity Therapy for Diabetes (1), the author's description in the discussion section, overlaps with the results section. In the discussion, what needs to be conveyed to readers is that bibliometric studies are conducted to examine the number of publications on a research topic, analyze the current state of research, and identify future potential and growth patterns (7). In keywords with citation burst, the keywords of “impaired glucose tolerance” had the highest strength, the author pointed out that many studies have revealed that lifestyle changes like physical activity or exercise efficacy can prevent the development of T2DM. Although impaired glucose tolerance (IGT) is a primary preventive indicator for diabetes, the relationship between exercise therapy and IGT has not been reported. Some studies have indicated that exercise therapy has a certain impact on IGT indicators (8, 9), the author suggested that “*Previous studies also revealed that higher intensities of exercise or adequate exercise may provide greater benefit for diabetes*,” which lacked supporting references. “*The key issue came with how much intensity or duration was enough to benefit health? What were the results of the health response of short-time with high-intensity exercise vs. long-time with low-intensity? As high intensity or vigorous exercise had a relatively higher risk of cardiovascular events, how to balance the benefit and risk overall for health?*” The author lacks further explanation to address these uncertainties, and only elaborates on the American Diabetes Association's recommendations for diabetes patients in the following sections, which is insufficient to fully address all aspects of the question posed. For example, Gao et al. (10) also raised the following issues in the discussion section of their article “*What can we learn from the inflammatory mechanism?*” However, they addressed these issues, supported their answers with relevant literature, and linked them to the article's theme with an in-depth study of the interaction mechanism between periodontitis and diabetes, while others have attempted to investigate the association between periodontitis and other systemic diseases, such as cardiovascular disease and obesity, through the host inflammatory response mechanism. Some consensus statements (11, 12) have established that participation in regular PA improves blood glucose control and can prevent or delay type 2 diabetes. A clinical study (13) presented details of the exercise therapies used in long-term studies, describing how the parameters for exercise prescriptions were applied in clinical practice. These parameters are described in terms of the frequency, intensity, duration, mode, and rate of progression of long-term therapeutic prescriptions.

In conclusion, for bibliometric research, we should not simply rely on the results obtained from software such as CiteSpace and Vosviewer, and make a simple record without analyzing the significance of these results. Although there are some doubts regarding the description of co-occurrence maps and the discussion section of this article, it is undeniable that the article objectively highlights the latest research frontiers in physical activity therapy for diabetes. More attention should be paid to physical activity therapy for patients with diabetes. In the future, diabetes treatment is not solely dependent on medication, but can also involve prevention through exercise, which can alleviate a range of diabetic complications.
